# Epidemiologic Relationship between Toscana Virus Infection and *Leishmania infantum* Due to Common Exposure to *Phlebotomus perniciosus* Sandfly Vector

**DOI:** 10.1371/journal.pntd.0001328

**Published:** 2011-09-20

**Authors:** Laurence Bichaud, Marc Souris, Charles Mary, Laëtitia Ninove, Laurence Thirion, Raphaël P. Piarroux, Renaud Piarroux, Xavier De Lamballerie, Rémi N. Charrel

**Affiliations:** 1 UMR 190, IRD-Université de la Méditerranée Aix-Marseille 2, Marseille, France; 2 Laboratoire de Parasitologie-Mycologie, AP-HM Timone, Marseille, France; National Institute of Allergy and Infectious Diseases, United States of America

## Abstract

Sand flies are recognised vectors of parasites in the genus *Leishmania* and a number of arthropod-borne viruses, in particular viruses within the genus *Phlebovirus*, family *Bunyaviridae*. In southern France, Toscana phlebovirus (TOSV) is recognized as a prominent cause of summer meningitis. Since Leishmania and TOSV have a common vector (*Phlebotomus perniciosus*), an epidemiologic link has been assumed for a long time. However, there is no scientific evidence of such a link between human leishmaniosis and phleboviral infections. To identify a possible link, we investigated the presence and distribution of antibodies against these two microorganisms (i) in individuals and (ii) at a spatial level in the city of Marseille (south-eastern France). Five hundred sera were selected randomly in the biobank of the Department of Parasitology of the Public Hospitals of Marseille. All sera were previously tested for IgG against Leishmania by Western Blotting, and TOSV IgG were detected by indirect immunofluorescence. The seropositivity rates were 21.4% for TOSV and 28% for Leishmania. Statistical analysis demonstrated that seropositivity for one pathogen was significantly associated with seropositivity to the other pathogen. This result provided the first robust evidence for the existence of an epidemiological relationship between *Leishmania infantum* and TOSV. Addresses of tested patients were geolocalized and integrated into Geographical Information System software, in order to test spatial relationship between the two pathogens. Spatial analysis did not allow to identify (*i*) specific patterns for the spatial distribution of positive serological results for TOSV or Leishmania, and (*ii*) a spatial relationship between Leishmania and TOSV positive serological results. This may reflect the fact that the sample studied was not powerful enough to demonstrate either a spatial clustering or co-location, *i.e.* that the actual risk exposure area is smaller than the mean of distance between patients in our study (245 m).

## Author Summary

Sand flies are tiny insects widely distributed in peri-Mediterranean countries where they can transmit Leishmania parasite and Toscana virus (TOSV). Since those microorganisms have a common vector, an epidemiologic link has been assumed for a long time. However, there is no scientific evidence of such a link between human leishmaniosis and phleboviral infections. To identify a possible link, we investigated the presence and distribution of antibodies to both Leishmania and TOSV in 500 sera of individuals living in Marseille, France. Antibodies are markers of past infections and reflect the exposition of population to pathogens. Statistical analysis demonstrated that seropositivity to one of these pathogens was significantly associated with seropositivity to the other pathogen. This result provided first robust evidence for an epidemiological relationship between *Leishmania infantum* and TOSV. Home addresses of tested patients were geolocalized in order to test spatial relationship between the two pathogens. This spatial analysis did not allow identifying either a spatial clustering or co-location, most probably because the sample was not powerful enough. Risk-factors of infection by TOSV (or Leishmania) would be investigated in seropositive patients' neighbourhood, in a range inferior to the mean of distance between patients in our study (245 meters).

## Introduction

Sand flies are tiny insects living in periurban or rural environments, often close to domestic animals and humans; they fly silently and only on short distances. Sand flies are widely distributed in peri-Mediterranean countries where their activity peaks during summertime. Their bite (only females are hematophagous) usually occurs at night [1]–[2].

Sand flies are recognised vectors of flagellate protozoan parasites in the genus *Leishmania*, causing diseases collectively known as leishmaniasis [3]. Zoonotic visceral leishmaniasis (ZVL) is caused by *Leishmania infantum* transmitted by phlebotomine flies from the vertebrate reservoir (dogs) to susceptible vertebrates (dogs and humans). ZVL has been described in China, Pakistan, Africa, Latin America and most countries of the Mediterranean region [4]. Its eco-epidemiology has been thoroughly studied particularly in south of France [5] where visceral leishmaniasis is more frequently reported than cutaneous infections [6].

Sandfly vectors can also transmit a number of arthropod-borne viruses within the families *Reoviridae* (Changuinola virus), *Rhabdoviridae* (Irririvirus, vesicular stomatitis virus, Isfahan virus) and *Bunyaviridae*. In the latter family, the viruses concerned are mostly 8 of the 9 viral species belonging to genus *Phlebovirus* [7]. In the Mediterranean area, several phleboviruses are circulating as demonstrated by virus isolation and/or molecular detection in sand flies, and some of them (*e.g.* Toscana virus, Naples virus and Sicilian virus) are recognised human pathogens [1].

Sicilian virus has been described during World War II in the corresponding region of Italy [8]. Toscana and Naples viruses belong to the species *Sandfly fever Naples virus* that currently includes also Tehran and Karimabad viruses, as well as newly recognized viruses such as Massilia, Punique and Algeria viruses.

In Italy, Southern France and Spain, molecular detection of phleboviruses suggested that human cases of infection are predominantly due to Toscana virus (TOSV) (which is the only phleboviral known pathogen identified to date in France). TOSV, first identified in Central Italy in the 1970′s [9]–[10], is recognized as a prominent cause of summer meningitis [11]. In France, both reports of infection cases [12], [13], [14] and seroprevalence studies have established that TOSV is circulating in the southeastern part of the country. It is likely that the incidence of TOSV infection is higher and that the occurrence of pauci-symptomatic forms or mild febrile illness occur more frequently than previously believed [15], [16].

Leishmania and TOSV have a common vector, *Phlebotomus perniciosus*. However, at the outset of this study, there was no scientific evidence for an epidemiologic link between the two microorganisms. In contrast to leishmaniasis, the natural history of phlebovirus infections is poorly known. Up to now, no animal reservoir of the virus has been identified and it is not likely that humans can play this role because of the short viremic period [8]–[17]. However, the maintenance of Toscana virus in sand fly by vertical transmission was demonstrated experimentally and corroborated by in natura observations showing a similar prevalence of infected male and female sand flies [18], [19].

Our hypothesis was that there is an epidemiological link between human leishmaniosis and phleboviral infections, since it is believed that they are both predominantly transmitted to humans via the bite of infected female *Phlebotomus perniciosus*. To identify this link, we investigated the presence and distribution of antibodies to both Leishmania and TOSV (i) in individuals and (ii) at a spatial level in the city of Marseille (south-eastern France) where human cases of visceral leishmaniasis [6] and TOSV infections [14] have been reported. Here, we present the results obtained and discuss their main implications.

## Materials and Methods

### Study area

The study area corresponded to the build-up area including Marseille (south-eastern France, on the Mediterranean coast) and its suburbs cities (∼250 km^2^). Geographical data suitable for Geographical Information System (GIS) are available on the website www.crige-paca.org.

### Serum samples

According to national regulations under the term of Biomedical Research (Huriet-Sérusclat law), patient's signature at the hospital entrance office warrants that all specimens collected during hospitalization for diagnostic purpose are accessible for research (excluding human genetic research) without specific consent of the patient. Five hundred sera, collected from patients hospitalized between 2001 and 2010 were selected randomly in the biobank of the department of Parasitology of the Public Hospitals of Marseille. All sera were previously tested for antibodies against Leishmania by Western Blotting [20]. Positive and negative results were noted L-IgG-POS and L-IgG-NEG, respectively.

Information on patient sex, age, home location and date of hospital admission was compiled to establish an anonymous database.

### Toscana virus IgG detection

IgG specific of TOSV were detected by indirect immunofluorescence (IIF) assay, as previously described [21], [22]. Sera were tested at 1∶20 dilution in phosphate-buffered saline. Blind reading was performed by two independent operators. Positive sera for Toscana virus were noted TOSV-IgG-POS and negative sera TOSV-IgG-NEG.

### GIS

Patients were geo-localized via home postal address (http://www.gpsfrance.net & http://maps.google.fr/). Geo-localized patients and the corresponding attributes (Identification number, sex, age, address, serological results) were integrated into a layer in the *SavGIS* GIS software (www.savgis.org/).

### Statistical analysis

Univariate analyses were performed to calculate statistics of positive patients for Leishmania and TOSV. Correlation between pathogens within an individual was searched out considering positive serology for one pathogen as an exposition factor and calculating statistics, relative risk, odd-ratio, and Pearson chi-square. Bivariate analyses (z-test, t-test) were performed to look for an effect of age on the serological status for Leishmania or TOSV. The influence of age on positive patients was searched out using non parametric test (Wilcoxon-Mann-Whitney test). To consider age of patient as a possible confounding factor, adjusted chi-square by Mantel-Haenszel method was calculated. All statistical analyses were performed using R software (http://fr.wikipedia.org/wiki/Site_Webwww.r-project.org) and *SavGIS* software.

### Spatial analysis

The global aim of the spatial analysis was to highlight possible relationships between environment and serology status or between serological statuses, considering spatial distribution of patients.

The spatial distribution of L-IgG-POS and TOSV-IgG-POS individuals was first mapped for visual interpretation. In order to study the overall position and dispersion of positive points, the mean center (MC) and the standard deviational ellipse (SDE) were calculated for each pathogen. To analyze the spatial clustering of points with positive serology (for Leishmania and TOSV independently), spatial statistical tests by simulation were performed. These tests used distances between nearest positive neighbors (DNN) and percentage of neighbors with same value (NSV). The result of the observed situation was compared to the distribution of results obtained by simulation using random distribution of positive points in the sample (Monte-Carlo simulation). Those analyses were also performed for points positive to both L-IgG and TOSV-IgG in order to test their spatial clustering or dispersion.

### Co-location analysis

A spatial co-location analysis was performed using a test based on Monte-Carlo simulations of a co-location index, in order to test the global spatial relationship between L-IgG-POS and TOSV-IgG-POS individuals [23]. The index is equal to the sum of the square roots of the distances between nearest neighbors' pairs (one L-IgG-POS point and one TOSV-IgG-POS point). Since the two sets of points that were compared corresponded to the same patients, it was necessary to assess that the results of the co-location test reflected only the possible correlation due to spatial relations, and did not take into account the possible correlation within a same individual. Thus, patients seropositive for both Leishmania and TOSV were taken into account for the calculation of the index, but not for the calculation of the variability of the index.

## Results

### TOSV seroprevalence

Of the 500 sera, a complete set of data could be obtained for 472 (m/f sex ratio: 1.30, median age: 47, range: 2–101). 28% of sera were positive for Leishmania and 21.4% had IgG antibodies against TOSV. Only 11% of sera presented IgG against both pathogens (Table 1).

**Table 1:** Results of serological analysis for Leishmania parasite and Toscana virus.

		TOSV-IgG		Total
		NEG	POS	
**L- IgG**	**NEG**	292	48	340
	**POS**	79	53	132
**Total**		371	101	472

Among positive sera for Leishmania, 40.2% were also positive for TOSV. In contrast, only 14.1% L-IgG-NEG sera were TOSV-IgG-POS. Individuals with antibodies to Leishmania were significantly more likely to be also seropositive for TOSV (RR = 2.84 [2.03 – 3.98] ; OR = 4.08 [2.57 – 6.48] ; χ2 = 38.32, p<0.001 ; all tests were significant with a very low p-value). In the same way, 52.5% of positive sera for TOSV were also positive for Leishmania. Considering positive serology for TOSV as an exposition factor, the risk to have antibodies against Leishmania was more than twice for TOSV-IgG-POS than for TOSV-IgG-NEG (RR = 2.46 [1.88 – 3.23]; OR = 4.08 [2.57 – 6.48] ; χ2 = 38.32, p<0.001 ; all tests were significant with a very low p-value).

**Figure 1:**
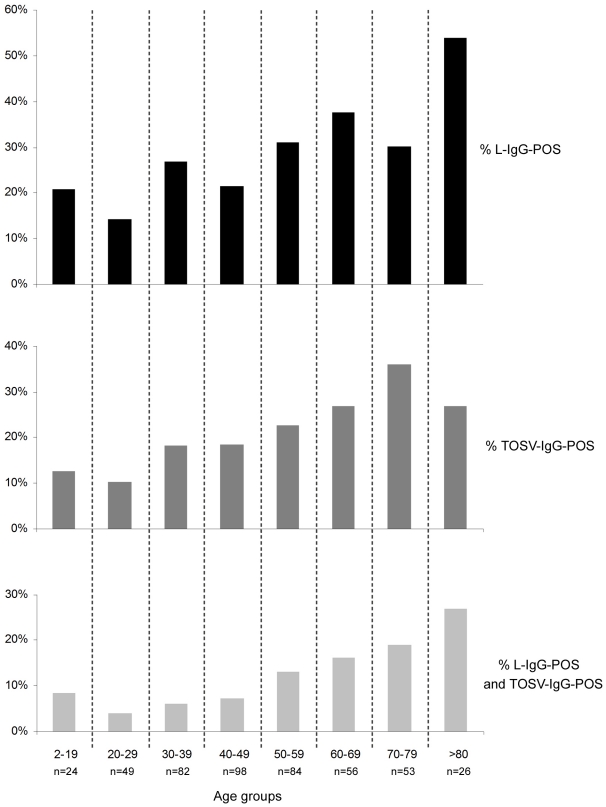
Evolution of Leishmania and TOSV seroprevalence in tested population according to age groups.

The age of patient had an effect on the serological status for Leishmania (z-test: p = 0.00035, t-test: p = 0.00034) and for TOSV (z-test : p = 0.00034, t-test : p = 0.00044) ; analyses showed a global increase of positivity with age (Figure 1). There was no significant difference between age of patients positive for Leishmania and for those positive for TOSV (WMW: p = 0.37). Considering age as a confounding factor, the link between both pathogens still existed (adjusted χ2 Mantel-Haenszel = 27.43, p<0.001).

### Spatial distribution of individuals tested

**Figure 2:**
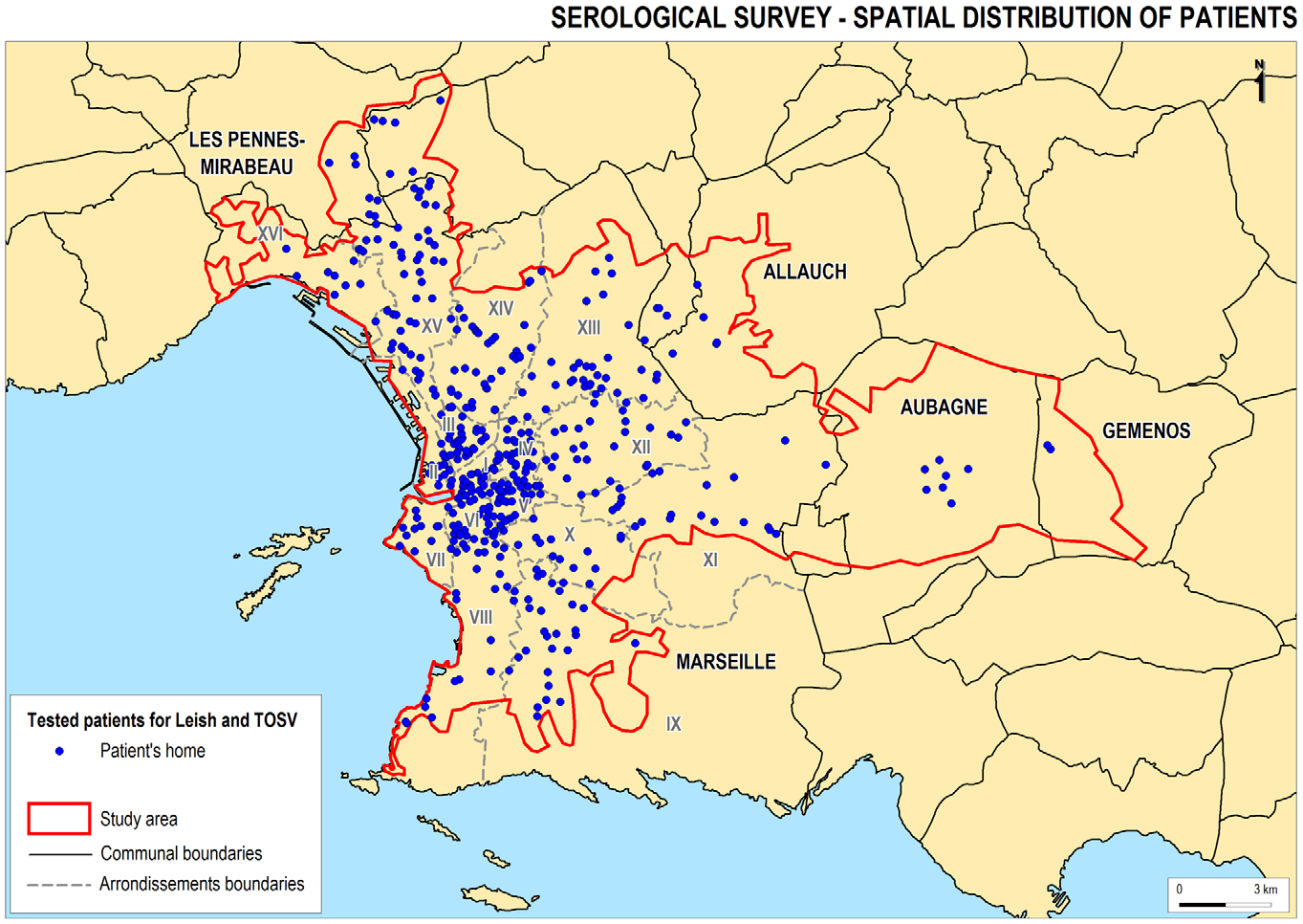
Spatial distribution of individuals tested in this study. Each point corresponds to a postal address. The distribution of the selected sample was found to follow the population density.

The spatial distribution of the selected sample (Figure 2) was found to follow the population density. The average of minimum distance between sample points was 245 m. Therefore, in the following spatial tests, we studied the spatial distribution of positive cases relative to the spatial distribution of all cases (the distribution of all points did not impact on the results of the tests).

### Spatial analysis

**Figure 3:**
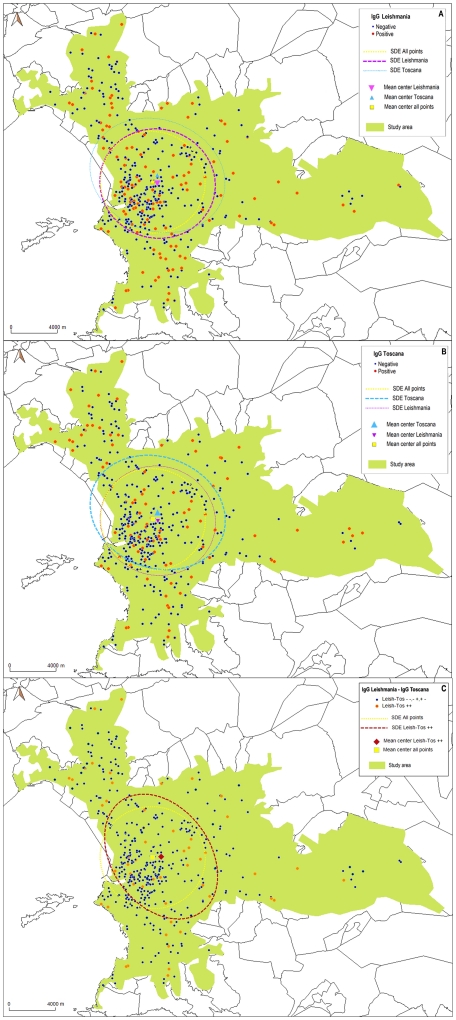
Analysis of spatial distribution of positive points. The maps represent the distribution of tested patients for Leishmania parasite (A), Toscana virus (B) and for patients positive for both pathogens (C). The orange dots represent positive sera. The ellipses represent the Standard Deviational Ellipses. The distribution of L-IgG-POS points, TOSV-IgG-POS points and points positive for both L-IgG and TOSV-IgG could not be distinguished from a random distribution amongst the sample tested.

The distribution of L-IgG-POS only points, TOSV-IgG-POS only points and points positive for both L-IgG and TOSV-IgG could not be distinguished from a random distribution amongst the sample tested (Figure 3A, 3B & 3C): the MC and the SDE calculated for these 3 categories of points did not show a specific localization of the spatial global position. The percentage of neighbours with the same value (NSV) and the distances between nearest positive neighbours (DNN) showed no significant difference between observed and simulated situations (Table 2): no clustering for any of these 3 categories of points could be detected.

**Table 2:** Results of analysis of the spatial clustering of positive points.

	NSV		DNN	
	Observed	Simulated	Observed	Simulated
L-IgG-POS points	30.3%	[16.3% – 39.4%]	557.6 m	[435.1 m – 626.6 m]
TOSV-IgG-POS points	28.71%	[8.7% – 33.7%]	636.7 m	[487.9 m – 756.2 m]
L-IgG-POS and TOSV-IgG-POS points	18.87%	[0% – 25%]	903.7 m	[622.7 m – 1195.8 m]

Positive points for Leishmania, for TOSV and for both pathogens were studied separately. No clustering could be detected: tests showed no difference between observed situations and simulated situations.

### Co-location analysis

When the intrinsic correlation was included (i.e. added to the calculation of the spatial correlation index and to the variability evaluation in the simulations), the test showed a significant spatial co-location between the two spatial patterns of positive sera: the mean of minimum distances observed between L-IgG-POS and TOSV-IgG-POS points (and vice-versa) was significantly lower than that expected in a random situation (Table 3).

**Table 3:** Results of the spatial co-location test including the intrinsic correlation.

Between points…	For α = 0,01 and m = 1		
	Mean of minimum distances (m)		p-value
	Observed	Simulated	
**L-IgG-POS and TOSV-IgG-POS**	224.3	[238–392]	0.001
**TOSV-IgG-POS and L-IgG-POS**	344	[349–512]	0.003

It was performed in order to test the global relationship between L-IgG-POS and TOSV-IgG-POS individuals. The test showed a significant spatial co-location between the two spatial patterns of positive sera.

However, when the intrinsic correlation was excluded from spatial correlation (*i.e.* the nearest positive neighbour of a positive point needed to be another object) and from variability calculation in simulations, the tests used showed no significant spatial co-location: the mean of minimum distances observed between points L-IgG-POS and points TOSV-IgG-POS was not different than that expected in a random situation (Table 4). The null hypothesis (H0: no co-location when intrinsic correlation is excluded) can not be rejected.

**Table 4:** Results of the spatial co-location test excluding the intrinsic correlation.

Between points…	For α = 0,01 and m = 1		
	Mean of minimum distances (m)		p-value
	Observed	Simulated	
**L-IgG-POS and TOSV-IgG-POS**	509	[411–588]	>0.05
**TOSV-IgG-POS and L-IgG-POS**	619	[506–670]	>0.05

It was performed in order to test the global relationship between L-IgG-POS and TOSV-IgG-POS individuals. When the intrinsic correlation was excluded from spatial correlation (*i.e.* the nearest positive neighbour of a positive point needed to be another object), the tests used showed no significant spatial co-location.

## Discussion

The spatial distribution of sand flies is the major recognized risk marker for leishmaniasis [24]: sand flies are considered as the unifying thread in the ecologic approach of leishmaniasis' study [25]. In France, two species are known to be vectors of *Leishmania infantum*: *Phlebotomus ariasi* and *Phlebotomus perniciosus* [5]. In the Marseille area, the predominant species is *P. perniciosus* that represents more than 96% of wild-caught sand flies [26] and has been identified for decades as the arthropod vector of zoonotic visceral leishmaniasis.

The serological panel examined here was not designed to provide a representative picture of the general population in the Marseille region nor to determine seroprevalence values. It was obviously biased since it was built from individuals initially tested for leishmaniasis. This may account for the seropositivity rates observed in this study for Toscana virus (21,4%) and leishmania (28%), *i.e.* higher than in previous studies in the same region (12% [15], and 16% [27], respectively), but did not hinder efforts to investigate the possible relationship between leishmaniasis and TOSV immunisation. The global increase of positivity with age observed in the studied population is in agreement with previous studies showing an age-dependent increase in TOSV-specific immunity (in Spain [28] and in Italy [29]) and in Leishmania-specific immunity [30].

Such analysis clearly indicated that seropositivity to one of these pathogens was significantly associated with seropositivity to the other pathogen. In the absence of any evidence for an immunological cross reactivity between leishmaniasis and TOSV, this indicates that persons exposed to TOSV infection are at greater risk of being infected with Leishmania parasite (and vice versa). This is compatible with our initial hypothesis that the epidemiological link between leishmaniasis and TOSV infection may be represented by the exposure to the bite of *Phlebotomus perniciosus*, their common arthropod vector.

The precise mechanism leading to double serological reactivity may rely either on the bite by sand flies co-infected by Leishmania and TOSV, or on successive bites by arthropods infected by a single pathogen. To date, no study has examined in the wild or experimentally the possible co-infection of sand flies by both microorganisms. Field studies in the Marseille area indicated that Leishmania- and TOSV-infected sand flies can co-circulate in a same site (Bichaud L., personal observations). However, the observed rates of sand flies infection for TOSV (∼0.29% in Marseille [26], 0.05% in Spain [28], 0.22% in Italy [9]) and those reported for *Leishmania infantum* (∼4% [31]) indicate a low probability of simultaneous double infection. Accordingly, it is unlikely that double immunization results from biting by co-infected sand flies, but rather that it is consecutive to successive infecting bites.

Based on the residence postal address, spatial analysis did not allow to identify *(i)* specific patterns for the spatial distribution of positive serological results for TOSV or Leishmania, and *(ii)* a spatial relationship between Leishmania and TOSV positive serological results. This may reflect the fact that the sample studied was not powerful enough to demonstrate neither a spatial clustering nor co-location, *i.e.* that the actual risk exposure area is smaller than the mean of distance between patients in our study (245 m). According to this hypothesis, risk-factors of infection by Toscana virus (or Leishmania parasite) have to be investigated in seropositive patients' neighbourhood, in a range inferior to 245 meters. This hypothesis holds with entomologic data demonstrating limited sandfly horizontal dispersion [32], [33], [34] and with the known mechanism of leishmaniasis in micro-foci [1].

However, it is also possible that exposure to sandfly bite has become progressively less dependent from the residence address. Urbanisation and improvement of sanitary conditions have presumably decreased the risk of night bite whilst new popular at-risk behaviors emerged, mainly linked to outdoor leisure activities such as horse-riding. There are 12 horse-ridding schools in the Marseille area and the highest sand flies density has been observed in stables and farms (Bichaud L., personal observations), where herbivore animals live and where sand flies are active during day light.

Leishmania parasite and phleboviruses are transmitted by the same vector, the principal reason why a possible epidemiological link has been assumed. Depending on the region of the world, the virus strain, the Leishmania type and the vector species vary; but sandfly fever and leishmaniasis often occur together because they share the same vector. The association of those diseases has long been known by tropical medicine specialists and medical entomologists working in southern Europe, North Africa and Central Asia. An example of this association, between Karimabad virus infection and cutaneous leishmaniasis, was reported in Iran [35]: ecology of both infections presents similar epidemiologic pattern (i.e. involvement of sand flies, gerbils and man). Therefore, the link established in this serological study between Toscana virus and *Leishmania infantum* is in agreement with previous knowledge.

Furthermore, ecological niches of sand flies within a community (town, forest or desert) are determined by the availability of larval breeding sites and vertebrate hosts and their distribution are quite focal. Since sandfly horizontal dispersion is known to be limited because of their short flight range, the risk of infection from sandfly-transmitted pathogens is also focal. Therefore, an epidemiologic link between two pathogens transmitted by sand flies, which overlap in their geographic distribution and share the same vector, would be possible.

Infections by TOSV and Leishmania represent an important public health problem in countries in which these microorganisms circulate. A proportion of the population ranging from 12–20% has serological evidence for past infection by TOSV in Mediterranean areas. In south-eastern France, 12% of the population had a history with TOSV, even though the clinical syndromes linked to TOSV is not fully elucidated and could be more diverse than believed [15]–[36]. TOSV has a tropism for the central nervous system and is a recognized prominent cause of summer meningitis and encephalitis. In Mediterranean countries, TOSV appears to be one of the three major viral pathogens (with enteroviruses and herpesviruses) involved in aseptic meningitis acquired during the summer [11]. In humans, infection with *Leishmania sp.* ranges from asymptomatic forms to severe visceral involvement. The most frequent clinical syndromes are visceral leishmaniasis. This disease has increased in importance in recent years because of its opportunist nature in HIV positive patients [37], [38] and is now considered as an emergent opportunist disease in southern Europe [39]. To date, no case of concomitant infection by TOSV and *Leishmania sp*., and no case of meningitis due to TOSV among patients with visceral leishmaniasis were reported. However, systematic testing is not performed and such situation is plausible. In this study, confirming the existence of an epidemiological relationship between *Leishmania infantum* and TOSV infections, serological analyses were retrospectively based on IgG detection for both Leishmania and TOSV. The presence of IgG indicates past infection but the date of infection remains unknown. To address this point and prove a possible co-infection, prospective cohort studies based on evidence of seroconversion, IgM detection or direct detection of each pathogen must be conducted.

In conclusion, this study performed in the region of Marseille, south-eastern France, was the first to provide robust evidence confirming the existence of an epidemiological relationship between *Leishmania infantum* and Toscana virus infections, presumably through the transmission by the common arthropod vector (*Phlebotomus perniciosus*). Further epidemiological and environmental studies, including entomological survey considering micro-foci, are requested to better estimate the population risk-factors of infection by Toscana virus. However this study also suggests that some of the epidemiological data available on Leishmaniasis may be used to decipher the epidemiology of Toscana virus infections.
